# A single‐institution pediatric and young adult interventional oncology collaborative: Novel therapeutic options for relapsed/refractory solid tumors

**DOI:** 10.1002/cam4.6026

**Published:** 2023-06-01

**Authors:** Raja Shaikh, Brent R. Weil, Christopher B. Weldon, Nan Chen, Wendy B. London, Morgan Krush, Megan Anderson, Mark Gebhardt, Alanna J. Church, Steven G. DuBois, Yana Pikman, Jennifer Spidle, Catherine B. Wall, Angela Feraco, Nicole J. Ullrich, Jennifer W. Mack, Elizabeth Mullen, Junne Kamihara, Suzanne Forrest, Suzanne Shusterman, Katherine A. Janeway, Ahmad Alomari, Horacio Padua, Carlos Rodriguez‐Galindo, Allison F. O'Neill

**Affiliations:** ^1^ Department of Radiology Boston Children's Hospital and Harvard Medical School Boston Massachusetts USA; ^2^ Department of Surgery Boston Children's Hospital and Harvard Medical School Boston Massachusetts USA; ^3^ Department of Pediatric Oncology Dana‐Farber Cancer Institute/Boston Children's Cancer and Blood Disorders Center and Harvard Medical School Boston Massachusetts USA; ^4^ Department of Pathology Boston Children's Hospital and Harvard Medical School Boston Massachusetts USA; ^5^ Department of Neurology Boston Children's Hospital and Harvard Medical School Boston Massachusetts USA; ^6^ Departments of Global Pediatric Medicine and Oncology St. Jude Children's Research Hospital Memphis Tennessee USA

**Keywords:** interventional, mRECIST, pediatric, radiology

## Abstract

**Background:**

Pediatric interventional oncology (PIO) is a growing field intended to provide additional or alternative treatment options for pediatric patients with benign or malignant tumors. Large series of patients treated uniformly and subjected to rigorous endpoints for efficacy are not available.

**Methods:**

We designed a collaborative initiative to capture data from pediatric patients with benign and malignant tumors who underwent a therapeutic interventional radiology procedure. Modified Response Evaluation Criteria in Solid Tumors (mRECIST) was utilized as a measure of radiologic response and data were collected regarding improvement in pain and functional endpoints. Cumulative incidence of progressive disease was calculated using both the treated site and the patient as the analytic unit.

**Findings:**

Forty patients, 16 with malignant tumors and 24 with benign tumors, underwent a total of 88 procedures. Cryo‐ and radiofrequency ablation were the most frequently utilized techniques for both cohorts of patients. A complete or partial response, or prolonged disease stability, were achieved in approximately 40% of patients with malignant tumors and 60% of patients with benign tumors. No patients had progressive disease as their best response. Resolution of pain and improved mobility with return‐to‐baseline activity were demonstrated across patients from both cohorts. Only minor complications were experienced.

**Interpretation:**

Interventional radiology‐guided interventions can serve as an alternative or complementary approach to the treatment of benign and malignant tumors in pediatric patients. Prospective, multi‐institutional trials are required to adequately study utility, treatment endpoints, and durability of response.

## Graphical abstract

Frequency of IO modalities utilized for the treatment of malignant (*n* = 33) (A) or benign (*n* = 55) (B) lesions in pediatric patients. Representative images demonstrating response of pediatric HCC to TACE (C, upper row pre‐treatment, lower row post‐treatment) and a pterygoid fossa desmoid to cryoablation (D, upper row pre‐treatment, lower row post‐treatment).

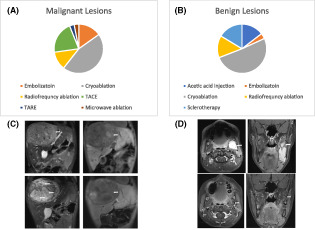

## 1 INTRODUCTION

Interventional radiology (IR), as a practice, describes the use of imaging to guide a minimally invasive procedural intervention. IR techniques have long been utilized in both pediatric and adult patients to obtain central venous or enteral access, image the vascular tree, obtain diagnostic tissue via needle biopsy, drain fluid collections, and facilitate genitourinary and gastroenterologic procedures.^1^, ^2^, ^3^ Over the last 10 years, the emerging field of Interventional Oncology (IO) has evolved to play a key role in the treatment of adult patients with benign or malignant tumors.^4^, ^5^ Examples of IO techniques include transarterial chemoembolization (TACE), transarterial radioembolization (TARE), radiofrequency ablation (RFA), microwave ablation (MWA), cryotherapy, and high‐intensity focused ultrasound (HIFU). The technique employed is selected based upon the technology available at a given institute, disease, tumor site, organ of origin, surrounding structures, and procedural intent.

For adults, and to a limited extent for children, IO‐guided therapies have proven to be a valuable adjunct to systemic therapy, a bridge to definitive local control, a tool to slow disease progression, and a mechanism by which to provide symptom relief.^6^, ^7^ The translation of IO methodologies to pediatric patients has been slow to evolve due to generally favorable upfront responses to systemic therapy and local control, limitations in the development and approval of new devices for use in children, pediatric patient size limitations, and a paucity of interventional radiologists trained in pediatrics.^8^ As such, little is known about the application of these techniques for pediatric patients beyond isolated reports or small series.

To systematically study IO procedures for pediatric patients, we created an IRB‐approved Pediatric IO (PIO) Collaborative to retrospectively collect technical and clinical data from patients with solid tumors undergoing IO procedures. The goal was to assess procedural intent, radiographic response, symptom improvement, and procedural complications for pediatric and young adult patients with solid tumors treated at the Dana‐Farber/Boston Children's Hospital Cancer and Blood Disorders Center. Herein, we describe our institutional experience as well as our approach to interpreting radiologic and functional outcomes.

## 2 MATERIALS AND METHODS

The eligible study population included pediatric, adolescent, and young adult patients from birth to 30 years of age treated with a PIO procedure for a benign or malignant solid tumor at Dana‐Farber/Boston Children's Hospital Cancer and Blood Disorders Center between January 2012 and February 2018. Data were collected from all patients discussed at a multidisciplinary solid tumor conference, attended weekly by key personnel from pediatric oncology, surgery, radiology, pathology, radiation oncology and IR, and deemed a suitable candidate for a PIO procedure. Optimal technique and timing for each procedure was agreed upon. Our institution offers TACE, TARE, RFA, MWA, bland embolization, and cryotherapy but not HIFU. All procedures were performed by pediatric trained and boarded interventional radiologists. Data regarding patient demographics, disease, procedural details, radiographic response, and symptoms was collected. Imaging studies were recommended at 1, 3, 6, and 12 months and performed either at Boston Children's Hospital or the patient's local institute and reviewed centrally. Follow‐up clinical information was sourced from proceduralist notes, clinical records, or communication with local providers. Patients with vascular malformations or retinoblastoma were excluded from this analysis given that care of these patients follows an alternative workflow.

Primary outcome measures included radiologic response and symptom and/or functional improvement. Radiologic response at the treated site was characterized utilizing modified RECIST (mRECIST) criteria, when feasible, with documentation of complete response (CR), partial response (PR), stable disease (SD), or progressive disease (PD).^9^ mRECIST was utilized given existing reports demonstrating optimal interpretation of response, following embolization or ablation in adult patients, utilizing this approach.^9^, ^10^, ^11^, ^12^, ^13^ When imaging studies were obtained but mRECIST criteria could not be applied or when imaging studies were not obtained, patient response was characterized as “unknown” and outcomes were described using functional endpoints alone. Functional endpoints for patients with initial symptoms included reduction in pain, enhanced mobility, improved activities of daily living (ADLs), and decreased swelling. As this data was sourced retrospectively, the following pain scoring scale was applied: (1) no improvement in pain, (2) mild/moderate improvement in pain, (3) significant improvement in pain. Changes in mobility, swelling, and ADLs were documented descriptively. Complications were graded utilizing the initial version of the Society of Interventional Radiology (SIR) criteria.^14^

Cumulative incidence of relapse, PD or regrowth were analyzed using (1) the treated lesion as the analytic unit; and (2) the patient as the analytic unit—the latter to account for competing PD at other non‐treated sites. For investigation of the treated lesion as the analytic unit, the time to event was calculated as the time from treatment of that lesion until the date of PD or regrowth at the lesion site; lesions without PD/recurrence were censored on the date of last imaging at which there was no PD or regrowth at the lesion site. For investigation of the patient as the analytic unit, the minimum time to event (PD or regrowth) across all sites and lesions was determined. Patients without a PD/recurrence event were censored on the date of last imaging at which there was no PD or regrowth. In each case, the cumulative incidence of relapse/PD/regrowth was calculated with adjustment for the competing risks of death and surgery/resection of the treated site. Evaluable patients for the analyses of radiologic response and cumulative incidence of relapse/PD/regrowth had at least one imaging follow‐up after treatment. For the purposes of this analysis, PR and SD were reported in aggregate given limited data on the optimal radiographic assessment for “success” of a procedure following intervention. CRs were reported separately. *p* < 0.05 was considered statistically significant. SAS Version 9.4 was utilized for all statistical analyses. The study was approved by the Institutional Review Board of the Dana‐Farber/Boston Children's Cancer and Blood Disorders Center (DFCI protocol 14‐289). Waiver of informed consent was granted given the retrospective nature of this data collection.

## 3 RESULTS

### 3.1 Patients and case demographics

**FIGURE 1:**
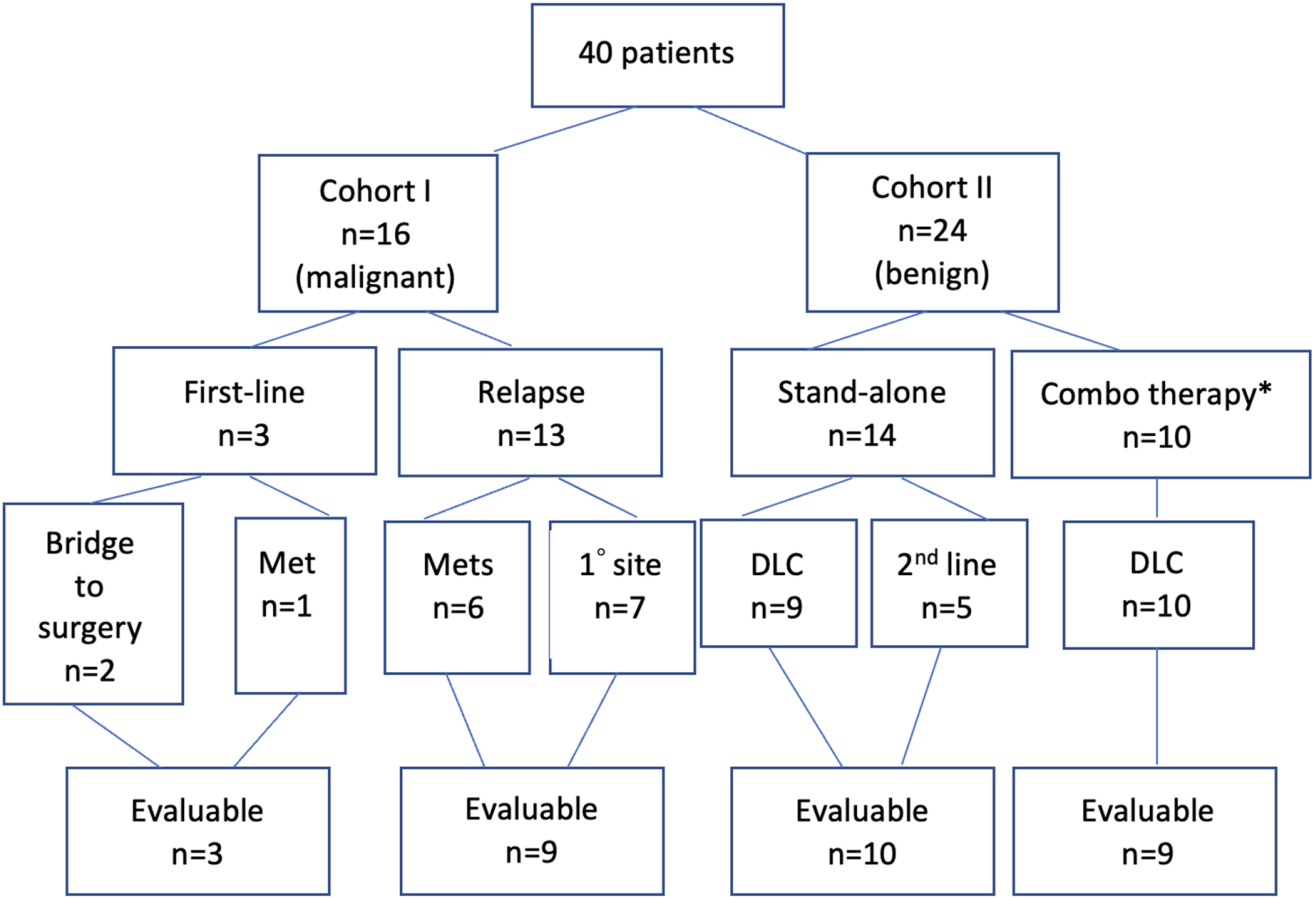
CONSORT diagram depicting patient cohort and evaluability. DLC, definitive local control.*All patients had a diagnosis of desmoid tumor. Evaluable = at least one imaging follow‐up after treatment.

During the six‐year study period, 40 patients underwent a total of 88 IO procedures (CONSORT Figure 1). Details regarding individual diagnoses, procedures, concomitant treatment, and follow‐up for all 40 patients can be found in Tables S1 and S2. In the complete cohort, 16 patients had malignant tumors and 24 had benign tumors. The malignant tumors most frequently treated were Ewing sarcoma, hepatocellular carcinoma (HCC), and osteosarcoma (OS). Thirteen patients (81%) had relapsed disease. Among these patients, six (46%) underwent IO therapies intended to treat metastatic sites in order to slow disease growth and/or mitigate pain. Of the remaining seven patients (54%) with relapsed disease, one underwent cryotherapy to a positive margin post‐hemipelvectomy, two underwent therapies to treat a local recurrence at the primary tumor site, and four underwent procedures targeting the primary site of disease despite the presence of metastatic disease elsewhere, with the goal to mitigate symptoms related to the primary tumor. The remaining three patients (19%) with malignant tumors, had PIO procedures performed as part of first‐line treatment: TACE as a bridge to definitive liver transplant for HCC, TACE and vessel embolization to mitigate bleeding and facilitate surgical resection of a glomus tumor, and RFA to metastatic gastrointestinal stromal tumor sites within the liver (Table S1).

**FIGURE 2:**
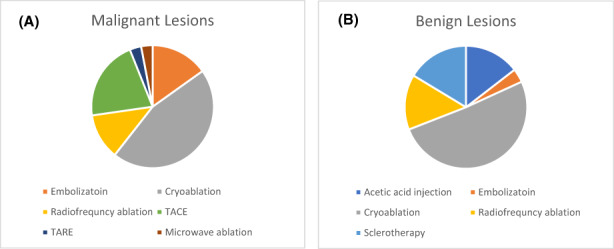
Frequency with which Interventional Oncology modalities were utilized for the treatment of malignant (*n* = 33) (A) or benign (*n* = 55) (B) lesions.

The benign tumor most frequently treated was desmoid tumor. All but two patients with desmoid tumor were receiving systemic therapy at the time of the PIO procedure or had recently concluded systemic therapy. Patients with benign tumors other than desmoid did not receive systemic therapy. Seven patients with non‐desmoid, benign tumors underwent a PIO procedure as definitive local control (DLC, Table S2). Twenty‐three of the 24 patients treated for a benign indication had unifocal disease; one patient with Gardner's syndrome had multifocal fibromatosis and more than one site was treated. The distribution of PIO procedures for each stratum is depicted in Figure 2. For each patient, the largest diameter of the treated tumor was recorded: the median (range) was 6.8 cm (0.7, 13.0 cm) and 5.3 cm (0.5, 15.2 cm) for patients with malignant (*n* = 16) versus benign (*n* = 24) disease, respectively.

### 3.2 Response evaluable population

Thirty‐one patients had serially imaged disease and were evaluable for the analyses of cumulative incidence of relapse/PD/regrowth and response (CONSORT Figure 1). The patient characteristics for this evaluable sub‐cohort are shown in Table 1. The median number of procedures performed was 2 (range: 1–5) for patients with both malignant and benign tumors (*p* = 0.55). The median follow‐up was 16 months (range: 1–86) and 36 months (range: 4–97) for patients with malignant and benign tumors, respectively (*p* = 0.15). When analyzing best response in the 12 patients with malignant tumors, three CRs were achieved for patients with malignant disease, and nine patients achieved either a PR or SD. Of the nine patients who initially achieved a PR or SD at the lesion level, five ultimately developed PD at the treated site (Table 2). Of the 19 patients with benign disease, 11 CRs were achieved, and eight patients achieved either a PR or SD. Four patients with benign disease ultimately developed PD at the treated site (Table 2). There was no difference in response rate for patients with malignant versus benign tumors (*p* = 0.14, Table 1). Figure S1 depicts radiographic disease response for three patients.

**TABLE 1:** Characteristics of patients with at least one imaging follow‐up after treatment (n = 31).

	Malignant (*n* = 12)	Benign (*n* = 19)	*p*‐Value
Demographics			
Age, years, median (range)	16 (2, 28)	13 (2, 22)	0.24^a^
Number of procedures, median (range)	2 (1, 5)	2 (1, 5)	0.55^a^
Follow‐up, months, median (range)	16 (1, 89)	36 (4, 97)	0.15^a^
Sex			
Male, *n* (%)	10 (83.3)	11 (57.9)	0.24^b^
Female, *n* (%)	2 (16.7)	8 (42.1)	
Site of origin			
Head/neck,^c^ *n* (%)	2 (16.7)	2 (10.5)	0.001^b^
Trunk,^d^ *n* (%)	10 (83.3)	5 (26.3)	
Extremities,^e^ *n* (%)	0	12 (63.2)	
Tissue of origin			
Bone, *n* (%)	2 (16.7)	6 (31.5)	0.067^b^
Soft tissue, *n* (%)	5 (41.6)	12 (63.2)	
Organ, *n* (%)	5 (41.6)	1 (5.3)	
Best response			
CR, *n* (%)	3 (25)	11 (57.9)	0.14^b^
PR/SD, *n* (%)	9 (75)	8 (42.1)	
PD, *n* (%)	0	0	
Symptoms			
No initial symptoms, *n* (%)	8 (66.7)	4 (21.0)	0.021^b^
Initial symptoms, *n* (%)	4 (33.3)	15 (79.0)	
Reduction in pain, *n* (%)	4 (100)	15 (100)	
Enhanced mobility, *n* (%)	0	10 (66.7)	
Improved ability to eat, *n* (%)	1 (25)	1 (6.7)	
Decreased swelling, *n* (%)	1 (25)	0	

^a^ Wilcoxon rank‐sum test was used to detect association of benign versus malignant for demographic and clinical continuous variables.

^b^ Fisher's exact test was used to test association of benign versus malignant for demographic and clinical categorical variables.

^c^ Head/neck include sites: mandible, neck, parapharygeal region.

^d^ Trunk include sites: kidney, abd wall, back, brachial plexus, iliac crest, shoulder, breast, iliac wing, acetabulum, liver, lung, hip/gluteal region, chest wall, epicardial node, paraspinal mass, pelvis, flank and abdominal wall.

^e^ Extremities include sites: calf, fibula, thigh, tibia, femur, leg, foot, arm, humerus, talus, lower extremity soft tissues.

**TABLE 2:** Crosstabulation of best response versus worst response for patients with malignant (*n* = 12) and benign (*n* = 19) tumors.

Malignant (*n* = 12)		Worst response		
		No PD	PD	Unknown
Best response	CR	3	0	0
	PR/SD	2	5	2
	Unknown	0	0	0

Benign (*n* = 19)		Worst response		
		No PD	PD	Unknown
Best response	CR	9	2	0
	PR/SD	6	2	0
	Unknown	0	0	0

**FIGURE 3:**
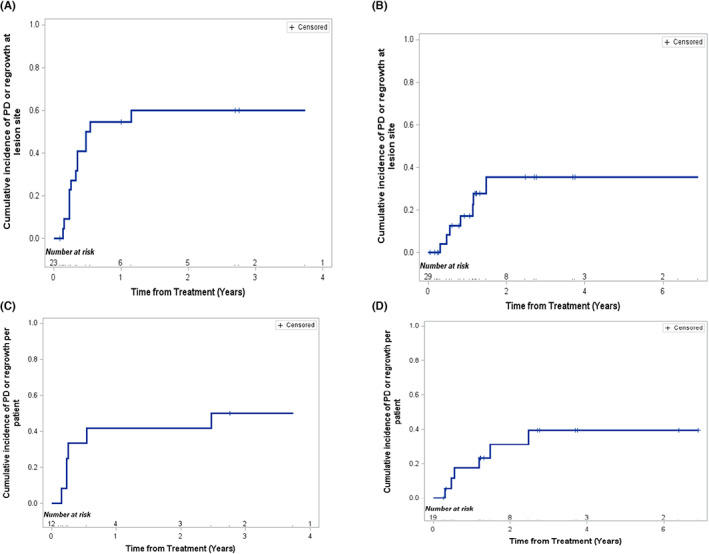
Cumulative incidence curves of progressive disease or regrowth at the lesion site, with adjustment for the competing risk of death or surgery/resection, in (A) malignant tumors (*n* = 23 lesions) or (B) benign tumors (*n* = 29 lesions). Cumulative incidence curve of PD or regrowth for patients with (C) malignant tumors (*n* = 12), or (D) benign tumors (*n* = 19) with adjustment for competing risk of death or surgery/resection.

Cumulative incidence curves were generated for the lesion as the analytic unit and also the patient as the analytic unit. With adjustment for the competing risk of death or surgery/resection, the 2‐year risk of developing PD or regrowth at the treated site was 60 ± 11% for patients with malignant tumors and 35 ± 12% for patients with benign tumors (Figure 3A,B). With adjustment for the competing risk of death or surgery/resection, the 2‐year risk for a patient to develop PD or regrowth, taking account disease at all sites, was 42 ± 15% for patients with malignant tumors and 34 ± 12% for patients with benign tumors (Figure 3C,D).

### 3.3 Functional endpoints

Of the image‐evaluable patients, 4/12 (33.3%) of patients with malignant tumors had initial symptoms while 15/19 (79.0%) with benign tumors had initial symptoms (*p* = 0.021). All patients with initially reported symptoms experienced an improvement in pain, activities of daily living, and swelling (Table 1, Supplementary Tables).

### 3.4 Procedural complications

The complete cohort of 40 patients were assessed for procedural complications. All patient with malignant tumors were hospitalized following their procedures for observation. Three patients experienced category A post‐procedural pain. Two patients had a prolonged admission for management of baseline tumor‐related pain, a third patient was admitted for a disease but not procedure‐related pneumothorax, and the patient with a glomus tumor went directly to surgery post‐embolization. Utilizing the SIR classification schema, all events fell into a mild category perceived predictable for the procedure being performed.^15^ Four patients with benign tumors experienced category A post‐procedural pain, swelling, or muscle spasm. One experienced category B pain after cryoablation for a right calf plexiform neurofibroma, and a third, experienced a category C peroneal nerve injury after cryotherapy for an aneurysmal bone cyst of the fibula. The patient with a peroneal nerve injury initially developed foot drop which recovered with physical therapy; he has residual decreased sensation over the dorsum of his foot. Utilizing the SIR classification schema, all events fell into a mild category perceived predictable for the procedure being performed.^15^

## 4 DISCUSSION AND CONCLUSIONS

We herein report on one of the largest single‐institution PIO experiences to date for patients with both malignant and benign tumors. Cryotherapy and RFA were the most frequently utilized techniques. Several CRs were achieved for patients with both malignant and benign tumors and no patients had PD as their best response. Patients with malignant disease did not experience overwhelming progression at untreated sites and still derived benefit from treatment of the designated site. For patients with initial symptoms, the procedure performed alleviated pain and/or contributed to improved function or quality of life in all cases. There were no significant procedural complications.

Publications describing PIO practice are limited to small case series. The largest meta‐analysis reporting on the role of PIO procedures in patients with cancer was recently published by Zambaiti et al. compiling data from 21 manuscripts reporting on a total of 329 procedures (TACE or RFA) in 286 patients with a median range of 12 years and a mean duration of follow‐up of approximately 1 year. Only six of the included studies demonstrated a statistically significant advantage with respect to operative time, mortality, and 2‐year tumor‐free survival for patients treated with neoadjuvant IR procedures.^16^ The second largest pediatric series surveyed data from 28 patients, with a median age of 9 years, undergoing 29 ablation procedures. Eighty‐six percent of cases were performed for an oncologic indication. The authors primarily report on technical endpoints noting significant limitations in collecting clinically annotated data.^17^ While limited by the lack of clinically relevant endpoints, these publications support the safety and tolerability of PIO procedures.^17^, ^18^, ^19^

There is existing literature regarding consensus use of PIO procedures to treat osteoid osteomas^20^, ^21^, ^22^ and accumulating experience regarding the treatment of pediatric patients with primary liver tumors or retinoblastoma.^23^, ^24^, ^25^, ^26^, ^27^, ^28^, ^29^, ^30^ Our single‐institution experience provides insight regarding additional patient cohorts to potentially pursue for future study. First, patients with desmoid tumors achieved disease control in 11/12 (92%) cases. As surgery has fallen out of favor for this tumor and as systemic medications provide variable benefit, this is a benign tumor for which PIO procedures should be studied prospectively. A more detailed technical report of our cohort of patients with desmoid tumors is forthcoming. Second, patients with relapsed/refractory bone tumors were treated with greatest frequency in the cohort of patients with malignant disease. Of the three patients treated with Ewing sarcoma, all achieved a PR at the treated site, one with moderate improvement in pain. Unfortunately all succumbed to systemic progression of disease. The third scenario warranting further study is lung nodule ablation, independent of disease histology. The two treated lung metastases in this series did not regrow. With the advent of more successful systemic therapies, PIO procedures in these patient cohorts may provide durable local control at sites not amenable to surgery, fewer delays in chemotherapy post‐procedure, and improved pain control sparing the sedating effects of systemic narcotics.

Our study demonstrates a successful collaboration between pediatric oncologists, surgeons, interventional radiologists, pathologists, radiologists, and statisticians—each of whom contributed to decisions surrounding type of procedure, interpretation of response, possible synergy with systemic therapies, procedure timing, and the appropriate statistical approach to capture clinical outcomes. The retrospective nature of this series introduces the following limitations: the type of procedure performed was at the discretion of the interventionalist, standard imaging sequences were obtained, and clinical notes and/or documented provider correspondence were relied upon for report of patient symptoms and impact on quality of life.

We conclude that a uniform PIO approach to treating patients with either malignant or benign tumors paired with prospectively collected, annotated patient data is crucial. This approach would allow for the study of exploratory endpoints including: long‐term procedural toxicities, application of more sophisticated imaging techniques, for example or pixel‐level radiomics or positron omission tomography,^31^ serial sample collection supporting correlative biology or a tissue‐level understanding of procedural effects on the tumor microenvironment,^32^ and patient‐reported outcomes research.^33^ Furthermore, understanding the role of PIO procedures in enhancing the efficacy of small molecule or immunotherapies would be of interest. Our hope is that this series will the support a future, multi‐institutional, uniform prospective study to validate the current findings. Such a study can pave the way for evidence‐based algorithms instructing the application of PIO procedures to advance pediatric oncology practice.

## AUTHOR CONTRIBUTIONS

**Raja Shaikh:** Conceptualization (lead); data curation (lead); formal analysis (equal); investigation (equal); methodology (equal); resources (equal); validation (equal); visualization (equal); writing – original draft (equal); writing – review and editing (equal). **Brent R Weil:** Conceptualization (equal); data curation (equal); formal analysis (equal); investigation (equal); methodology (equal); validation (equal); writing – original draft (equal); writing – review and editing (equal). **Christopher B Weldon:** Conceptualization (equal); investigation (equal); methodology (supporting); validation (supporting); writing – original draft (supporting); writing – review and editing (supporting). **Nan Chen:** Data curation (lead); formal analysis (lead); methodology (lead); validation (equal); writing – original draft (equal); writing – review and editing (equal). **Wendy B London:** Data curation (lead); formal analysis (lead); methodology (lead); validation (equal); writing – original draft (equal); writing – review and editing (equal). **Morgan Krush:** Data curation (equal); resources (equal); software (equal); writing – review and editing (equal). **Megan E Anderson:** Conceptualization (equal); data curation (equal); writing – review and editing (equal). **Mark Gebhardt:** Conceptualization (equal); data curation (equal); writing – review and editing (equal). **Alanna J Church:** Conceptualization (equal); data curation (equal); writing – review and editing (equal). **Steven DuBois:** Conceptualization (equal); investigation (equal); methodology (equal); writing – review and editing (equal). **Yana Pikman:** Conceptualization (equal); investigation (equal); methodology (equal); writing – review and editing (equal). **Jennifer Spidle:** Resources (equal); writing – review and editing (equal). **Catherine B. Wall:** Resources (equal); writing – review and editing (equal). **Angela M. Feraco:** Conceptualization (equal); investigation (equal); methodology (equal); writing – review and editing (equal). **Nicole Ullrich:** Conceptualization (equal); investigation (equal); methodology (equal); writing – review and editing (equal). **Jennifer W. Mack:** Conceptualization (equal); investigation (equal); methodology (equal); writing – review and editing (equal). **Elizabeth A. Mullen:** Conceptualization (equal); investigation (equal); methodology (equal); writing – review and editing (equal). **Junne Kamihara:** Conceptualization (equal); investigation (equal); methodology (equal); writing – review and editing (equal). **Suzanne Forrest:** Conceptualization (equal); investigation (equal); methodology (equal); writing – review and editing (equal). **Suzanne Shusterman:** Conceptualization (equal); investigation (equal); methodology (equal); writing – review and editing (equal). **Katherine Janeway:** Conceptualization (equal); investigation (equal); methodology (equal); writing – review and editing (equal). **Ahmad I. Alomari:** Investigation (equal); project administration (equal); resources (equal); writing – review and editing (equal). **Horacio Padua:** Investigation (equal); project administration (equal); resources (equal); writing – review and editing (equal). **Carlos Rodriguez‐Galindo:** Conceptualization (equal); data curation (equal); formal analysis (equal); investigation (equal); methodology (equal); resources (equal); validation (equal); writing – original draft (equal); writing – review and editing (equal). **Allison O'Neill:** Conceptualization (equal); data curation (equal); formal analysis (equal); funding acquisition (equal); investigation (equal); methodology (equal); resources (equal); supervision (equal); validation (equal); writing – original draft (equal); writing – review and editing (equal).

## CONFLICT OF INTEREST STATEMENT

The authors report no conflicts of interest.

## Supporting information


**Figure S1:** Images depicting treatment of pediatric conventional HCC with TACE (3A), a fibrolamellar HCC lung nodule with RFA (3B), and a pterygoid fossa desmoid with cryoablation (3C). (3A): Coronal T2‐weighted fat suppressed MR images demonstrate a large conventional hepatocellular carcinoma (upper left) which heterogeneously enhances on T1‐weighted contrast enhanced MR imaging (upper right). After TACE, the T2‐weighted fat suppressed MR images demonstrate significant necrosis (bottom left) and lack of contrast enhancement on coronal T1‐weighted contrast enhanced MR images (bottom right). The graph on the right demonstrates alpha‐fetoprotein (AFP) decline after two rounds of systemic cisplatin/doxorubicin (red arrow) and after each round of TACE (black arrows). (3B): Axial CT imaging demonstrates a focal lesion in the right lower lobe (left) with scarring from resolution post‐RFA of the lesion (right). (3C): From left to right: axial and coronal T1‐weighted contrast MR images demonstrate an avidly enhancing desmoid tumor in the left pterygoid fossa pre‐treatment (first two images) and complete resolution on follow‐up axial and coronal T1‐weighted contrast enhanced MR images post‐cryoablation (second two images).

**Table S1:** Patients treated for malignant disease.

**Table S2:** Patients treated for benign disease.

## Data Availability

Data is available upon reasonable request.
