# Supplementary material for: A single‐institution pediatric and young adult interventional oncology collaborative: Novel therapeutic options for relapsed/refractory solid tumors
Source: Cancer Med. 2023 Jun 1;12(12):13300–8. doi: 10.1002/cam4.6026 (PMC10315804; doi:10.1002/cam4.6026)

Supplemental Figure 1

Images depicting treatment of pediatric conventional HCC with TACE (3A), a fibrolamellar HCC lung nodule with RFA (3B) and a pterygoid fossa desmoid with cryoablation (3C).
3A: Coronal T2-weighted fat suppressed MR images demonstrate a large conventional hepatocellular carcinoma (upper left) which heterogeneously enhances on T1-weighted contrast enhanced MR imaging (upper right). After TACE, the T2-weighted fat suppressed MR images demonstrate significant necrosis (bottom left) and lack of contrast enhancement on coronal T1-weighted contrast enhanced MR images (bottom right). The graph on the right demonstrates alpha-fetoprotein (AFP) decline after two rounds of systemic cisplatin/doxorubicin (red arrow) and after each round of TACE (black arrows). **3B**: Axial CT imaging demonstrates a focal lesion in the right lower lobe (left) with scarring from resolution post-RFA of the lesion (right). **3C**: From left to right: axial and coronal T1-weighted contrast MR images demonstrate an avidly enhancing desmoid tumor in the left pterygoid fossa pre-treatment (first two images) and complete resolution on follow-up axial and coronal T1-weighted contrast enhanced MR images post-cryoablation (second two images).

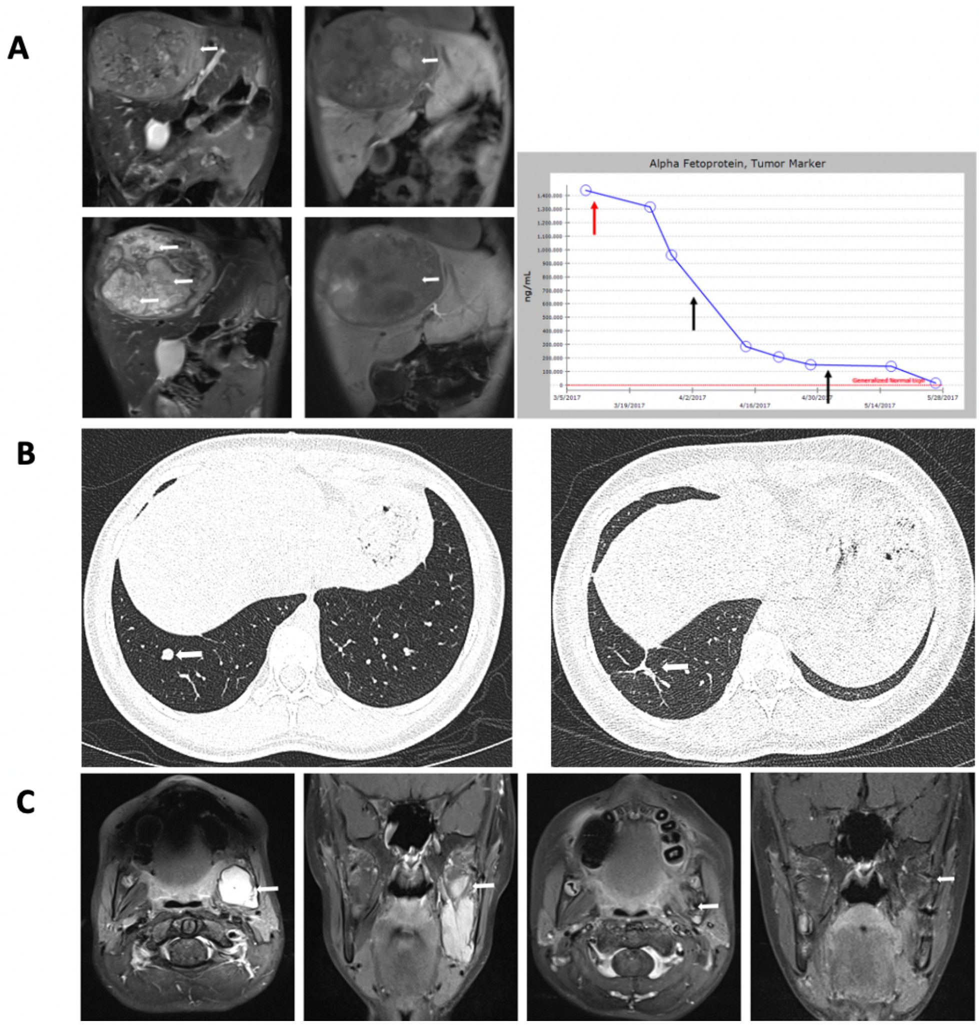

Supplement: Supplementary file 1 — Figure S1: Images depicting treatment of pediatric conventional HCC with TACE (3A), a fibrolamellar HCC lung nodule with RFA (3B), and a pterygoid fossa desmoid with cryoablation (3C). (3A): Coronal T2‐weighted fat suppressed MR images demonstrate a large conventional hepatocellular carcinoma (upper left) which heterogeneously enhances on T1‐weighted contrast enhanced MR imaging (upper right). After TACE, the T2‐weighted fat suppressed MR images demonstrate significant necrosis (bottom left) and lack of contrast enhancement on coronal T1‐weighted contrast enhanced MR images (bottom right). The graph on the right demonstrates alpha‐fetoprotein (AFP) decline after two rounds of systemic cisplatin/doxorubicin (red arrow) and after each round of TACE (black arrows). (3B): Axial CT imaging demonstrates a focal lesion in the right lower lobe (left) with scarring from resolution post‐RFA of the lesion (right). (3C): From left to right: axial and coronal T1‐weighted contrast MR images demonstrate an avidly enhancing desmoid tumor in the left pterygoid fossa pre‐treatment (first two images) and complete resolution on follow‐up axial and coronal T1‐weighted contrast enhanced MR images post‐cryoablation (second two images). [file CAM4-12-13300-s002.pdf]
