# Supplementary material for: A single‐institution pediatric and young adult interventional oncology collaborative: Novel therapeutic options for relapsed/refractory solid tumors
Source: Cancer Med. 2023 Jun 1;12(12):13300–8. doi: 10.1002/cam4.6026 (PMC10315804; doi:10.1002/cam4.6026)
Supplement: Supplementary file 2 — Table S1: Patients treated for malignant disease. [file CAM4-12-13300-s001.docx]

Supplementary Table 1. Patients treated for Malignant Disease

| Patient | Age (yrs) | Dx | Stage (L = local, M = metastatic R = relapse) | Systemic tx (R = recent, C = concomittent) | Site | IO Intervention | # proce-dures | Response by lesion (mRECIST) | Complications (Y/N, type, grade) | Improvement in pain (1=none, 2 = mild/moderate, 3 = significant) | QOL | Life Status |
| --- | --- | --- | --- | --- | --- | --- | --- | --- | --- | --- | --- | --- |
| 1 | 13 | BCOR-CCNB3 | L (R) | VIT (C) | R acetabulum | Cryoablation | 1 | Unkwn% | Y, pain (A) | N/A* | N/A* | DOD |
|  |  |  |  |  |  |  |  |  |  |  |  |  |
| 2 | 17 | HCC | M (R) | Pembro (C) | Liver | Y-90 | 1 | PR | Y, pain (A) | 3 | Appetite | DOD |
|  |  |  |  |  |  |  |  |  |  |  |  |  |
| 3 | 16 | RMS | L (R) | Oral CP, XRT (R) | Parapharyngeal | Cryoablation | 4 | PR | N | 2 | N/A* | DOD |
|  |  |  |  |  |  |  |  |  |  |  |  |  |
| 4 | 20 | OS | M (R) | Nivo/ipi (R),  CP, avastin, sorafenib (C) | L femur | Cryoablation | 1 | Unkwn | N | N/A* | N/A* | DOD |
|  |  |  |  |  |  |  |  |  |  |  |  |  |
| 5 | 15 | HCC | M (R) | Pembro (C) | Lung | RFA | 1 | CR | N | N/A@ | N/A@ | AWRD |
|  |  |  |  |  |  |  |  |  |  |  |  |  |
| 6 | 28 | MPNST | L (R) | Dox, olaratumab (R) | R Hip/Gluteus | Cryoablation | 1 | PR | N | 1 | Swelling | DOD |
|  |  |  |  |  |  |  |  |  |  |  |  |  |
| 7 | 15 | HCC | M (R) | Pembro (C) | Lung | RFA | 1 | CR | N | N/A@ | N/A@ | DOD |
|  |  |  |  |  |  |  |  |  |  |  |  |  |
| 8 | 15 | EWS | M (R) | VIT (R) | Chest wall | Cryoablation | 1 | PR | N | 2 | N/A* | DOD |
|  |  |  |  |  |  |  |  |  |  |  |  |  |
| 9 | 18 | HCC | M (R) | Tem/Thal (C) | Epicardial node | Cryoablation | 2 | PR | N | N/A@ | N/A@ | DOD |
|  |  |  |  |  |  |  |  |  |  |  |  |  |
| 10 | 17 | OS | M (R) | CP, avastin, soafenib, XRT (R) | Paraspinal mass | Cryoablation | 1 | Unknwn | N | N/A* | N/A* | DOD |
|  |  |  |  |  |  |  |  |  |  |  |  |  |
| 11 | 20 | EWS | M (R) | Avastin (C) | Pelvis | TACE (Cisplat) | 3 | PR | N | N/A* | N/A* | DOD |
|  |  |  |  |  |  |  |  |  |  |  |  |  |
| 12 | 16 | OS | M (R) | Sorafenib (C) | R lower extremity (soft tissues) | Cryoablation | 1 | Unknwn | N | N/A* | N/A* | DOD |
|  |  |  |  |  |  |  |  |  |  |  |  |  |
| 13 | 4 | Glomus | M | None | L neck | TACE (Bland x2), Embo | 3 | PR& | N | N/A@ | N/A@ | AWRD |
|  |  |  |  |  |  |  |  |  |  |  |  |  |
| 14 | 2 | HCC | L | None | Liver | TACE (Dox) | 2 | PR& | Y, pain (A) | N/A@ | N/A@ | NED |
|  |  |  |  |  |  |  |  |  |  |  |  |  |
| 15 | 13 | GIST | M | Sorafenib (C) | Liver | RFA, Embo | 7 | CR/PR | N | N/A@ | N/A@ | AWRD |
|  |  |  |  |  |  |  |  |  |  |  |  |  |
| 16 | 27 | EWS | M (R) | None | L flank, Abd wall | Cryoablation | 3 | PR | N | N/A* | N/A* | DOD |

BCOR-CCNB3: fusion-positive sarcoma, HCC: hepatocelluar carcinoma, RMS: rhabdomyosarcoma, OS: osteosarcoma, MPNST: malignant peripheral nerve sheath tumor, GIST: gastrointestinal stromal tumor, Y-90: Yttrium-90, RFA: radiofrequency ablation, TACE: transarterial chemoembolization, Embo: bland embolization, DOD: Died of disease, AWRD: alive with residual disease

VIT: vincristine, irinotecan, temozolomide, Pembro: pembrolizumab, Dox: doxorubicin, Cisplat: cisplatin, Tem/Thal: temodar, thalidomide, XRT: external beam radiotherapy.

N/A*: Insufficient follow-up for reassessment of pain/QOL post-procedure

N/A@: Aymptomatic at disease site, no impact on QOL

%: Treated positive margins s/p hemipelvectomy

&: Bridge to surgery
