# Supplementary material for: A single‐institution pediatric and young adult interventional oncology collaborative: Novel therapeutic options for relapsed/refractory solid tumors
Source: Cancer Med. 2023 Jun 1;12(12):13300–8. doi: 10.1002/cam4.6026 (PMC10315804; doi:10.1002/cam4.6026)
Supplement: Supplementary file 3 — Table S2: Patients treated for benign disease. [file CAM4-12-13300-s003.docx]

Supplementary Table 2. Patients treated for Benign Disease

| Patient # | Age (yrs) | Tumor | Stage (L = local, M = mutifocal, R = relapse) | Systemic tx (R = recent, C = concomittent) | Site | IO Intervention | # procedures | Response by lesion (  mRECIST) | Complications (Y/N, type, grade) | Improvement in pain (1=none, 2 = mild/moderate, 3 = significant) | QOL | Follow-up | Life Status |
| --- | --- | --- | --- | --- | --- | --- | --- | --- | --- | --- | --- | --- | --- |
| 1 | 9 | PN | L | None | R calf | Cryoablation | 1 | SD | Y, pain (A), edema | 3 | Ambulatory | 76 mths | AWRD |
|  |  |  |  |  |  |  |  |  |  |  |  |  |  |
| 2 | 8 | Desmoid | L | Dox (C) | L pterygoid fossa | Cryoablation | 2 | CR | N | 2 | Ability to eat  Trismus | 14 mths | NED |
|  |  |  |  |  |  |  |  |  |  |  |  |  |  |
| 3 | 13 | ABC | L | None | L fibula | Sclerotherapy, Embo, Cryo | 5 | PR/CR | Y, peroneal nerve injury (C) | 3 | Ambulatory | 45 mths | NED |
|  |  |  |  |  |  |  |  |  |  |  |  |  |  |
| 4 | 19 | Desmoid | L | None | R thigh | Cryoablation | 1 | CR | Y, muscle spams (A) | 3 | Ambulatory | 3 mths | NED |
|  |  |  |  |  |  |  |  |  |  |  |  |  |  |
| 5 | 14 | CBT | L | None | L talus | RFA | 1 | Unknwn | N | 3 | Full activity | 46 mths | NED |
|  |  |  |  |  |  |  |  |  |  |  |  |  |  |
| 6 | 16 | OO | L | None | L tibia | RFA | 1 | Unknwn | N | 3 | Full activity | 22 mth | NED |
|  |  |  |  |  |  |  |  |  |  |  |  |  |  |
| 7 | 18 | GCT | L | None | L femur | RFA | 2 | PR | N | 2 | N/A | 14 mths^ | NED% |
|  |  |  |  |  |  |  |  |  |  |  |  |  |  |
| 8 | 9 | OO | L | None | R femur | RFA | 1 | Unknwn | N | 3 | Full activity | 34 mth | NED |
|  |  |  |  |  |  |  |  |  |  |  |  |  |  |
| 9 | 12 | AML | L | None | L kidney | Embo | 1 | CR | N | N/A@ | N/A@ | 32 mths | NED |
|  |  |  |  |  |  |  |  |  |  |  |  |  |  |
| 10 | 13 | Desmoid | L | Sorafenib (R) | R post thigh | Cryoablation | 1 | PR | Y, swelling (A) | 2 | Full activity | 6 mths^ | AWRD |
|  |  |  |  |  |  |  |  |  |  |  |  |  |  |
| 11# | 13 | Desmoid | M | VA (R) | Abd wall, back | Cryoablation | 3 | Unkwn | N | N/A* | N/A* | 6* | DOD |
|  |  |  |  |  |  |  |  |  |  |  |  |  |  |
| 12 | 14 | Desmoid | L | Sorafenib (C) | R brachial plexus | Cryoablation | 2 | CR | Y, pain (B) | 2 | Use of arm | 29 mths^ | AWRD |
|  |  |  |  |  |  |  |  |  |  |  |  |  |  |
| 13 | 14 | Desmoid | L | Sorafenib (C) | R post iliac crest | Cryoablation | 2 | CR | N | 3 | N/A* | 6 mths^ | NED& |
|  |  |  |  |  |  |  |  |  |  |  |  |  |  |
| 14 | 22 | Desmoid | L | Sorafenib (R) | L leg | Cryoablation | 2 | CR | Y, pain/swelling (A) | 2 | N/A* | 17 mths^ | AWRD |
|  |  |  |  |  |  |  |  |  |  |  |  |  |  |
| 15 | 20 | Desmoid | L | None | L foot | Cryoablation | 2 | CR | N | 1 | N/A* | 33 mths^ | AWRD |
|  |  |  |  |  |  |  |  |  |  |  |  |  |  |
| 16 | 13 | Desmoid | L | VB/MTX (R) | R shoulder | Acetic acid, Cryoablation | 4 | PR | N | N/A* | N/A* | 5 mths^ | AWRD |
|  |  |  |  |  |  |  |  |  |  |  |  |  |  |
| 17 | 15 | GCT | L | None | R talus | Cryoablation, RFA | 2 | CR | N | 3 | Full activity | 4 mths | NED |
|  |  |  |  |  |  |  |  |  |  |  |  |  |  |
| 18 | 21 | GCT | L | None | R femur | RFA | 1 | CR | N | 3 | Full activity | 44 mths | NED |
|  |  |  |  |  |  |  |  |  |  |  |  |  |  |
| 19 | 2 | Desmoid | L | Hydroxyurea (R),  Doxil (C), VB/MTX (C) | R neck | Cryoablation | 3 | CR | N | N/A@ | N/A@ | 3 mth^ | AWRD& |
|  |  |  |  |  |  |  |  |  |  |  |  |  |  |
| 20 | 20 | FA | L | None | L breast | Cyroablation | 1 | Unknwn | N | 2 | N/A@ | 7 mths | NED |
|  |  |  |  |  |  |  |  |  |  |  |  |  |  |
| 21 | 6 | Desmoid | L | Hydroxyurea (C) | R arm | Acetic acid | 4 | SD | N | 2 | Activity  A | 99 mths | AWRD |
|  |  |  |  |  |  |  |  |  |  |  |  |  |  |
| 22 | 16 | Desmoid | L | Sorafenib (R) | L shoulder | Cryoablation | 3 | CR | N | N/A* | N/A* | 14 mths | AWRD |
|  |  |  |  |  |  |  |  |  |  |  |  |  |  |
| 23 | 13 | ABC | L | None | L iliac wing | Sclero, Cryo | 4 | CR | N | 3 | Ambulatory  A | 15 mths | NED |
|  |  |  |  |  |  |  |  |  |  |  |  |  |  |
| 24 | 11 | CBT | L | None | R humerus | RFA | 2 | PR/CR | N | 2 | Activity  A  (mild) | 14 mths | AWRD |
|  |  |  |  |  |  |  |  |  |  |  |  |  |  |

PN: plexiform neuroblastoma, ABC: aneurysmal bone cyst, CBT: chondroblastoma, OO: osteoid osteoma, GCT: giant cell tumor, AML: renal angiomyolipoma, FA: fibroadenoma, Y-90: Yttrium-90, RFA: radiofrequency ablation, TACE: transarterial chemoembolization, Embo: bland embolization, DOD: Died of disease, AWRD: alive with residual disease, NED: no evidence of disease

Dox: doxorubicin, VA: vincristine/actinomycin, VB/MTX: vinblastine/methotrexate

N/A*: Insufficient imaging available, insufficient follow-up for reassessment of pain/QOL post-procedure

N/A@: Aymptomatic at disease site, no impact on QOL

#: germline APC gene mutation

&: eventual surgery

%: eventual curettage

^: Eventual start on systemic therapy
